# Factors associated with influenza vaccine uptake among healthcare workers in Georgia, 2021–2024

**DOI:** 10.1371/journal.pone.0356495

**Published:** 2026-08-25

**Authors:** Oksana Artemchuk, Giorgi Chakhunashvili, Iris Finci, Madelyn Rojas, Esther Kissling, Khatuna Zakhashvili, Mark A. Katz, Lia Sanodze

**Affiliations:** 1 World Health Organization Regional Office for Europe, Copenhagen, Denmark; 2 National Center for Disease Control and Public Health, Tbilisi, Georgia; 3 Epiconcept, Paris, France; Universitas Syiah Kuala, INDONESIA

## Abstract

**Background:**

Healthcare workers (HCWs) are prioritised to receive influenza vaccine annually in Georgia. We aimed to identify factors associated with influenza vaccine uptake among Georgian HCWs.

**Methods:**

We used self-reported influenza vaccination data for three influenza seasons (2021–2024) and 2023 survey data on attitudes towards influenza from a prospective cohort of HCWs in six hospitals in Georgia. HCWs who received influenza vaccine in at least one season were compared with those who were not vaccinated in any season. Adjusted odds ratios (aORs) and 95% confidence intervals (CIs) were calculated using multivariable logistic regression to identify factors associated with vaccination uptake.

**Findings:**

Of 403 participants, the median age was 46 (IQR 36–54), 364 (90%) were female, 168 (42%) were nurses or midwives, 85 (21%) were support staff, 75 (19%) were doctors, and 75 (19%) were other staff. Overall, 289 (72%) received influenza vaccine in at least one season, while 114 (28%) did not receive it in any season. Multivariable analysis showed no differences in vaccine uptake by sex or occupation. HCWs ≥ 30 years were more likely to be vaccinated, with those aged 40–49 having the highest odds (aOR 2.67, 95% CI 1.09–6.59). HCWs who did not believe the vaccine was safe (aOR 0.27, 95% CI 0.11–0.68), those who doubted its effectiveness (aOR 0.36, 95% CI 0.14–0.89), and HCWs at Hospital B (aOR 0.06, 95% CI 0.02–0.23) were less likely to be vaccinated.

**Conclusions:**

Georgian HCWs who questioned influenza vaccine safety and effectiveness, and those at one hospital, had lower odds of vaccination over three years, whereas older HCWs were more likely to be vaccinated. Future campaigns to promote influenza vaccination among HCWs should address safety and effectiveness concerns and target demographic disparities.

## Introduction

Healthcare workers (HCWs) are at increased risk for influenza infection. Influenza vaccination remains the best intervention to prevent influenza infection. The World Health Organization (WHO) considers HCWs to be a priority group for annual influenza vaccination [1].

Influenza vaccination provides direct protection to HCWs by reducing the risk of symptomatic illness and severe complications [2,3]. Vaccinated HCWs are also less likely to transmit influenza to patients, thereby lowering the risk of nosocomial infections, particularly among vulnerable populations such as the elderly or immunocompromised [4,5]. Furthermore, higher vaccination coverage among HCWs has been associated with reduced absenteeism during influenza seasons, supporting continuity of care and mitigating strain on healthcare services [6,7].

However, annual influenza vaccine uptake among HCWs varies widely across countries and regions. A recent meta-analysis reported an overall global influenza vaccination rate of 41.7% among HCWs. Regional coverage ranged from 67.1% in the Americas (USA, Canada, Peru and Costa Rica) to 6.5% in Africa (Morocco, Egypt and Sierra Leone) [8].

HCWs’ attitudes, perceptions, and confidence in vaccines influence vaccination decisions [9]. Understanding factors associated with influenza vaccination uptake among HCWs, including attitudes and perceptions about influenza vaccine, may help inform targeted efforts to promote vaccine uptake among HCWs.

In recent years, variability in vaccination uptake has increasingly been examined through the concept of vaccine hesitancy, defined as delay in acceptance or refusal of vaccines despite availability of vaccination services [10]. Vaccine hesitancy is a complex and context-specific phenomenon influenced by factors such as confidence in vaccine safety and effectiveness, perceived risk of disease, and access to vaccination services [10,11].

Studies conducted during the COVID-19 pandemic demonstrated that vaccine hesitancy also occurs among HCWs and is associated with sociodemographic characteristics, profession, trust in health authorities, and previous vaccination behaviour, including influenza vaccination history [12–14].

More recent reviews continue to highlight the importance of vaccine confidence, perceived safety, and institutional trust as key determinants of vaccination behaviour among HCWs [15–17].

Georgia is an upper-middle income country in the South Caucasus region with a population of 3.7 million people. The Georgia Ministry of Health recommends influenza vaccination for HCWs [18]. In 2019, influenza coverage among HCWs in Georgia reported annually through the WHO/UNICEF Joint Reporting Form on Immunization was 64% [19].

From 2021–2024, the National Center for Disease Control and Public Health, Georgia (NCDC) together with WHO Regional Office for Europe conducted a prospective cohort study among HCWs in six Georgian hospitals to measure COVID-19 and influenza vaccine effectiveness (VE) [20]. We used data from this VE study with the aim of evaluating sociodemographic factors and attitudes about influenza vaccine associated with influenza vaccine uptake in at least one of the three seasons (2021–2022, 2022–2023, and 2023–2024) among Georgian HCWs.

## Methods

### Study design and study population

Methods related to the COVID-19 and influenza vaccine effectiveness study in Georgian HCWs have been previously described [21]. Briefly, in early 2021, we invited all HCWs at six hospitals in Georgia to enrol in a longitudinal prospective cohort study to evaluate COVID-19 and influenza vaccine effectiveness. Of the total of 3849 HCWs across the six participating hospitals, 1606 (42%) were enrolled in the study.

The six participating hospitals are secondary level facilities located in urban areas of Georgia and include clinical and referral hospitals providing inpatient medical care.

### Data collection

We administered questionnaires about attitudes towards influenza infection and vaccination at study enrolment (early 2021), and again one and two years later, during re-enrolment for Year 2 and Year 3 (early 2022 and early 2023).

Influenza vaccination status in the 2021–2022, 2022–2023 and 2023–2024 influenza epidemic season was self-reported by participants. We defined the influenza epidemic season as epidemiological week 40 of one calendar year through week 20 of the following year [22].

For our analysis, we used a dataset that only included data from HCWs who participated in all 3 years of the study. We further restricted our dataset to only include participants who had completed the sociodemographic questionnaire in the first year of the study, the influenza vaccination status questions in all three study seasons, and the questions about attitudes towards influenza and influenza vaccine in the Year 3 (2023) re-enrolment questionnaire.

We restricted the analysis to participants who completed all three years of follow-up because the outcome variable required information on influenza vaccination status across the three influenza seasons (2021–2022, 2022–2023, and 2023–2024), and key attitudinal variables were collected in the Year 3 questionnaire. Including only participants with complete vaccination history and attitudinal data allowed us to classify vaccination uptake across the entire three-year study period and evaluate associations with attitudes measured at the end of follow-up.

In addition, given the modest sample size of participants with complete three-year follow-up data, modelling vaccination uptake separately by season would have substantially reduced statistical power and limited multivariable analyses. Therefore, we focused on overall uptake across the study period.

The Year 3 re-enrolment survey included 4 questions about attitudes towards influenza and influenza vaccine where participants were asked to provide answers on a Likert scale, a common measurement method used in research to evaluate attitudes, opinions and perceptions. The Year 3 re-enrolment survey also included questions about reasons for receiving or not receiving the influenza vaccine in the 2022–2023 influenza epidemic season. For the purposes of the analysis, we grouped possible responses into the following binary variables: Strongly agree/Mildly agree and Neutral/Mildly disagree/Strongly disagree; Extremely effective/Very effective/Somewhat effective and Not too effective/Not at all effective; Extremely worried/Very worried/Moderately worried and A little worried/Not at all worried.

Study data were uploaded and stored in the REDCap system (Research Electronic Data Capture, Vanderbilt University, Nashville, Tennessee, USA). The data for analysis were accessed on 25 March 2023. Authors had no access to information that could identify individual participants during or after data collection.

### Statistical analysis

We described the study population overall and by socio-demographic, clinical and work-related characteristics. We also described the population by their influenza vaccination status, in two groups: 1) not vaccinated in any of the three study seasons and 2) vaccinated in at least one of the three study seasons.

We conducted univariable and multivariable logistic regression analyses to identify independent factors associated with receipt of influenza vaccine in at least one of the three study seasons.

Our primary exposures of interest were age, sex, hospital of work, chronic medical conditions, occupation, smoking status, household size, providing hands-on medical care to patients, having regular contact with patients, performing aerosol-generating procedures, self-rated health status, department of work, attitudes towards influenza vaccination.

We first conducted univariable and partially adjusted logistic regression analyses to examine associations between influenza vaccination status and potential covariates. In these partially adjusted models, each covariate was assessed while adjusting for age, sex, occupation, and hospital. Variables with a p-value < 0.05 in these models were considered for inclusion in the multivariable logistic regression model. Age, sex, occupation, and hospital were included in the multivariable model a priori, based on theoretical relevance and previous literature.

In the multivariable analysis, we used backward stepwise elimination to remove covariates not significantly associated with the outcome (p > 0.05). Variables were removed sequentially, beginning with the least significant (i.e., those with the smallest effect sizes), until only statistically significant variables remained. After each removal, a likelihood ratio test was performed to assess the significance of the change in model fit. Only variables that significantly contributed to the model (p < 0.05) were retained in the final model. A priori variables (age, sex, occupation, and hospital) were retained in all models regardless of statistical significance, to adjust for potential confounding and ensure robust estimation of the exposure effect. We presented adjusted odds ratios (aORs) and corresponding 95% confidence intervals (CIs) from the final model.

### Sensitivity analysis

As a sensitivity analysis, we restricted the outcome to influenza vaccination uptake during the 2023–2024 influenza epidemic season, when attitudinal variables were measured. We included all participants with available Year 3 data and applied the same univariable and multivariable logistic regression approach as in the primary analysis.

All analyses were conducted using Stata version 16.0 (StataCorp LLC, College Station, TX, USA).

### Ethics

The study was approved by the Georgia NCDC Institutional Review board (IRB # 021-014) and the Ethical Review Boards of WHO (reference number CERC.0097B). All participants provided written informed consent.

## Results

### Characteristics of participants

Overall, of the 1606 HCWs who were enrolled in Year 1, 403 (25%) participants had complete data, were still enrolled in Year 3, and were included in our analysis. The median age was 46 (IQR = 36–54), 364 (90%) were female, 168 (42%) were nurses or midwives, 85 (21%) were support or janitorial staff, 75 (19%) were doctors and 75 (19%) were other unspecified staff. A third of HCWs [134 (33%)] reported having at least one underlying condition, 131 (33%) reported smoking currently or in the past, and 289 (72%) reported their self-rated health status as “excellent,” “very good,” or “good” (Table 1).

**Table 1:** Sociodemographic characteristics of HCWs (N = 403) overall and by influenza vaccination status, Georgia, 2021–2024.

Characteristics	Total study population (N = 403)	Not vaccinated in any of the three study seasons (n = 114; 28%)	Received influenza vaccine in at least one of the three study seasons (n = 289; 72%)
**Hospital study site**			
Hospital A	28 (7%)	4 (4%)	24 (8%)
Hospital B	63 (16%)	49 (43%)	14 (5%)
Hospital C	127 (31%)	25 (22%)	102 (35%)
Hospital D	56 (14%)	7 (6%)	49 (17%)
Hospital E	129 (32%)	29 (25%)	100 (35%)
**Age, years**	46 (IQR: 36–54)	43 (IQR: 33–54)	48 (IQR: 37–55)
**Age groups, years**			
18–29	53 (13%)	22 (19%)	31 (11%)
30–39	80 (20%)	26 (23%)	54 (19%)
40–49	109 (27%)	24 (21%)	85 (29%)
50–59	108 (27%)	33 (30%)	75 (26%)
60+	53 (13%)	9 (8%)	44 (15%)
**Sex**			
Female	364 (90%)	106 (93%)	258 (89%)
Male	39 (10%)	8 (7%)	31 (11%)
**Occupation** ^**a**^			
Doctors	75 (19%)	13 (11%)	62 (21%)
Nurses or midwives	168 (42%)	46 (40%)	122 (42%)
Support staff or Janitorial staff	85 (21%)	26 (23%)	59 (20%)
Other staff	75 (19%)	29 (25%)	46 (16%)
**Has regular contact with patients** ^**b**^			
No contact	84 (21%)	26 (23%)	58 (20%)
Has contact	319 (79%)	88 (77%)	231 (80%)
**Provides hands-on medical care to patients**			
No	193 (48%)	62 (54%)	131 (45%)
Yes	210 (52%)	52 (46%)	158 (55%)
**Household size** ^**c**^			
Lives alone	83 (21%)	25 (22%)	58 (20%)
Lives with family members	320 (79%)	89 (78%)	231 (80%)
**Underlying conditions** ^**d**^			
None	269 (67%)	87 (76%)	182 (63%)
One or more	134 (33%)	27 (24%)	107 (37%)
**Smoking history**			
Never smoked	272 (67%)	84 (74%)	188 (65%)
Former and current smokers	131 (33%)	30 (26%)	101 (35%)
**Performs aerosol-generating procedures**			
Do not perform	246 (61%)	71 (62%)	175 (61%)
Perform	157 (39%)	43 (38%)	114 (39%)
**Self-rated health status**			
Excellent/Very good/Good	289 (72%)	87 (76%)	202 (70%)
Fair/Poor	108 (27%)	27 (24%)	81 (28%)
**Department of work**			
Inpatient/Outpatient units/services	322 (80%)	88 (77%)	234 (81%)
Office/Housekeeping wards	81 (20%)	26 (23%)	55 (19%)
**Season of influenza vaccination**			
Did not receive a vaccine in the 2021–2022 season	245 (61%)	114 (100%)	131 (45%)
Received a vaccine in the 2021–2022 season	158 (39%)	0 (0%)	158 (55%)
Did not receive a vaccine in the 2022–2023 season	212 (53%)	114 (100%)	98 (34%)
Received a vaccine in the 2022–2023 season	187 (47%)	0 (0%)	187 (66%)
Did not receive a vaccine in the 2023–2024 season	180 (45%)	114 (100%)	66 (23%)
Received a vaccine in the 2023–2024 season	216 (55%)	0 (0%)	216 (77%)

Note:

^a^Support staff includes radiology technicians, lab specialists, pharmacists, biologists. Janitorial staff includes cleaning and laundry workers, kitchen staff, social workers, drivers, and security officers. Unspecified types of occupations were included in other staff category.

^b^Includes contact with the following patients: infants aged <1 year, children aged 1–12 years, teenagers aged 13–19, adults aged 20–64, older adults aged 65 and older, and pregnant women.

^c^Living with family members include from 1 up to 5 family members.

^d^One or more underlying conditions include: cancer, chronic heart disease, high blood pressure/hypertension, chronic kidney disease, chronic liver disease, chronic lung disease, diabetes, immunocompromised, neurological disease, obesity, and autoimmune disorder.

^e^Aerosol-generating procedures include: collect a respiratory specimen or sputum specimen, administer a nebuliser, apply nasal cannula, oxygen face mask, or mechanical ventilation, perform tracheal intubation, manual ventilation, suction of fluids or secretions, chest physiotherapy, or bedside bronchoscopy.

Most participants reported having regular contact with patients [319 (79%)] and providing hands-on medical care to patients [210 (52%)]. Less than half of participants reported performing aerosol-generating procedures [157 (39%)]. Most participants worked in inpatient or outpatient units/services [322 (80%)], and most participants [320 (79%)] reported living with family members. Overall, 289 (72%) participants received influenza vaccine in at least one of the 3 seasons, while 114 (28%) participants were never vaccinated against influenza in any season. Of the 289 participants who reported having received influenza vaccine in at least one of the 3 seasons, 81/289 (28%) had received vaccine in all 3 seasons, 110/289 (38%) received vaccine in two seasons and 98/289 (34%) received vaccine in only one season.

### Attitudes towards influenza vaccination

Most participants [351 (87%)] said they thought that influenza vaccine was “extremely effective” or “very effective” or “somewhat effective” in preventing influenza, and most participants [357 (89%)] “strongly” or “mildly” agreed that influenza vaccination was safe. More than three-quarters of participants [320 (79%)] were not worried about becoming ill with influenza during the next 12 months. The large majority [373 (93%)] of HCWs strongly or mildly agreed with the statement, “If I am fully vaccinated against influenza, I will be less likely to miss work because of becoming ill with influenza” (Table 2).

**Table 2:** Attitudes towards influenza vaccination overall and by influenza vaccination status, Georgia, 2021–2024.

Attitudes	Total study population (403; 100%)	Not vaccinated in any of the three study seasons (114; 28%)	Received influenza vaccine in at least one of the three study seasons (289; 72%)
**Influenza vaccine is safe**			
Strongly agree/Mildly agree	357 (89%)	85 (24%)	272 (76%)
Neutral/Mildly disagree/Strongly disagree	46 (11%)	29 (63%)	17 (37%)
**Influenza vaccination is effective**			
Extremely effective/Very effective/Somewhat effective	351 (87%)	79 (23%)	272 (77%)
Not too effective/Not at all effective	52 (13%)	35 (67%)	17 (33%)
**How worried are you about getting sick with influenza during the next 12 months?**			
Extremely worried/Very worried/Moderately worried	83 (21%)	24 (29%)	59 (71%)
A little worried/Not at all worried	320 (79%)	90 (28%)	230 (72%)
**If I am fully vaccinated against influenza, I will be less likely to miss work because of getting sick with influenza**			
Strongly agree/Mildly agree	373 (93%)	103 (28%)	270 (72%)
Neutral/Mildly disagree/Strongly disagree	30 (7%)	11 (37%)	19 (63%)

In the univariable analysis we evaluated 14 sociodemographic, occupational, and attitudinal variables. Of these, 7 variables met criteria to be included in the multivariable analysis, of which 4 remained significant in the multivariable analysis (Table 3).

**Table 3:** Univariable and partially adjusted analysis of factors associated with uptake of influenza vaccine in at least one of the three study seasons among health care workers, Georgia, 2021–2024.

Factors	Adjusted Odds Ratio (aOR) 95%CI	Overall p-value
**Hospital**		0.000
Hospital A	Ref	
Hospital B	0.05 (0.02-0.18)	
Hospital C	0.74 (0.23-2.38)	
Hospital D	1.17 (0.30-4.52)	
Hospital E	0.55 (0.17-1.77)	
**Age group (years)**		0.186
18–29	Ref	
30–39	2.13 (0.89-5.07)	
40–49	2.60 (1.13-6.02)	
50–59	2.14 (0.94-4.90)	
60+	3.00 (1.05-8.51)	
Sex		0.327
**Female**	Ref	
Male	1.60 (0.63-4.09)	
**Occupation** ^**a**^		0.402
Doctors	Ref	
Nurses/midwives	0.97 (0.43-2.16)	
Support staff / Janitorial staff	0.71 (0.30-1.66)	
Other staff	0.57 (0.24-1.35)	
**Providing hands-on medical care to patients**		0.730
No	Ref	
Yes	1.15 (0.52-2.56)	
**Household size** ^**b**^		0.504
Live alone	Ref	
Live with family members	1.24 (0.66-2.34)	
**Influenza vaccine is safe**		0.007
Strongly agree/Mildly agree	Ref	
Neutral/Mildly disagree/Strongly disagree	0.15 (0.07-0.31)	
**Influenza vaccination is effective**		0.000
Extremely effective/Very effective/Somewhat effective	Ref	
Not too effective/Not at all effective	0.17 (0.08-0.36)	
**How worried are you about getting sick with influenza during the next 12 months?**		0.903
Extremely worried/Very worried/Moderately worried	Ref	
A little worried/Not at all worried	1.04 (0.54-1.99)	
**If I am fully vaccinated against influenza, I will be less likely to miss work because of getting sick with influenza**		0.012
Strongly agree/Mildly agree	Ref	
Neutral/Mildly disagree/Strongly disagree	0.34 (0.14-0.79)	
**Comorbidities** ^**c**^		0.148
None	Ref	
One or more	1.55 (0.86-2.18)	
**Smoking status**		0.352
Never smoked	Ref	
Former smoker/Current smoker	1.36 (0.71-2.61)	
**Perform aerosol-generating procedures** ^**d**^		0.587
No	Ref	
Yes	0.82 (0.39-1.70)	
**Self-rated health status**		0.422
Excellent/Very good/Good	Ref	
Fair/Poor	0.78 (0.42-1.44)	
**Department of work**		0.651
Inpatient/Outpatient wards	Ref	
Office/Housekeeping wards	1.22 (0.51-2.92)	
**Have a regular contact with patients** ^**e**^		0.652
No contact	Ref	
Has contact	0.84 (0.39-1.80)	

^a^Support staff includes radiology technicians, lab specialists, pharmacists, biologists. Janitorial staff includes cleaning and laundry workers, kitchen staff, social workers, drivers, and security officers. Unspecified types of occupations were included in other staff category.

^b^Living with family members include from 1 up to 5 family members.

^c^One or more underlying conditions include: cancer, chronic heart disease, high blood pressure/hypertension, chronic kidney disease, chronic liver disease, chronic lung disease, diabetes, immunocompromised, neurological disease, obesity, and autoimmune disorder.

^d^Aerosol-generating procedures include: collect a respiratory specimen or sputum specimen, administer a nebuliser, apply nasal cannula, oxygen face mask, or mechanical ventilation, perform tracheal intubation, manual ventilation, suction of fluids or secretions, chest physiotherapy, or bedside bronchoscopy.

^e^Includes contact with the following patients: infants aged <1 year, children aged 1–12 years, teenagers aged 13–19, adults aged 20–64, older adults aged 65 and older, and pregnant women.

Compared to HCWs aged 18–29 years, HCWs in the age group 40–49 years were more likely to get vaccinated (adjusted odds ratio (aOR): 2.67, 95% CI 1.09–6.59). While older HCWs in other age group categories were also more likely to have received influenza vaccine, the aORs were not statistically significant (Table 4).

**Table 4:** Multivariable analysis of factors associated with uptake of influenza vaccine in at least one of the three study seasons among health care workers, Georgia, 2021–2024.

Factors	Adjusted Odds Ratio (aOR) 95%CI	p-value
**Hospital**		
Hospital A	Ref	
Hospital B	0.06 (0.02-0.23)	0.000
Hospital C	1.02 (0.30-3.44)	0.978
Hospital D	1.07 (0.27-4.30)	0.919
Hospital E	0.59 (0.17-1.97)	0.388
**Age group (years)**		
18–29	Ref	
30–39	1.98 (0.79-4.97)	0.144
40–49	2.67 (1.09-6.59)	0.032
50–59	2.17 (0.89-5.24)	0.086
60+	2.86 (0.94-8.69)	0.063
**Influenza vaccination is effective**		
Extremely effective/Very effective/Somewhat effective	Ref	
Not too effective/Not at all effective	0.36 (0.14-0.89)	0.027
**Influenza vaccine is safe**		
Strongly agree/Mildly agree	Ref	
Neutral/Mildly disagree/Strongly disagree	0.27 (0.11-0.68)	0.006
**Sex**		
Female	Ref	
Male	1.66 (0.63-4.36)	0.305
**Occupation** ^**a**^		
Doctors	Ref	
Nurses/midwives	1.14 (0.50-2.90)	0.751
Support staff / Janitorial staff	0.90 (0.37-2.53)	0.825
Other staff	0.56 (0.23-1.36)	0.207

^a^Support staff includes radiology technicians, lab specialists, pharmacists, biologists. Janitorial staff includes cleaning and laundry workers, kitchen staff, social workers, drivers, and security officers. Unspecified types of occupations were included in other staff category.

HCWs who did not believe influenza vaccine was safe (aOR: 0.27, 95% CI 0.11–0.68), HCWs who thought that influenza vaccine was not effective (aOR: 0.36, 95% CI 0.14–0.89), and HCWs who worked in Hospital B (aOR: 0.06, 95% CI 0.02–0.23) were less likely to get vaccinated.

In a sensitivity analysis restricted to influenza vaccination uptake during the 2023–2024 season (the year in which attitudes were measured), perceived vaccine safety and perceived effectiveness in preventing missed work remained strongly associated with vaccination uptake. Institutional differences were also observed. Results were consistent with the primary analysis (S1 Table).

### Reasons for getting and not getting influenza vaccine in 2022–2023 season

HCWs who did not get vaccinated in the 2022–2023 season, cited as the main reason for not getting vaccinated “even if I get the flu, it won’t be severe, so I don’t need vaccination” [97 (66%)] followed by “I do not trust the official recommendations” [84 (58%)] (Table 5). Among HCWs who did get vaccinated, the main reason reported was “to protect my health” [211 (99%)] (Table 6).

**Table 5:** Reasons for not getting influenza vaccine, Georgia, 2022–2023.

Reasons	Yes, n (%)	No, n (%)
I am afraid of the side effects of the vaccine (n = 150)	47 (31%)	103 (69%)
I think that the vaccine will not protect me from infection (n = 147)	79 (54%)	68 (46%)
I don’t think the vaccine will protect me from severe illness (n = 151)	48 (32%)	103 (68%)
If we weight the risks and benefits of vaccination the risks outweigh the benefits (n = 145)	41 (28%)	104 (72%)
I’m unlikely to get the flu, so I don’t need a vaccination (n = 151)	77 (51%)	74 (49%)
Even if I get the flu, it won’t be severe, so I don’t need vaccination (n = 146)	97 (66%)	49 (34%)
I am against vaccination in general (n = 145)	24 (17%)	121 (83%)
I prefer natural immunity (after infection) to immunity formed as a result of vaccination (n = 147)	75 (51%)	72 (49%)
I do not trust the official recommendations (n = 146)	84 (58%)	62 (42%)
I was advised not to get the flu shot (n = 146)	13 (8.9%)	133 (91%)
I was not informed that I had to get a flu shot (n = 145)	1 (0.7%)	144 (99%)
I wanted to get vaccinated, but I didn’t have enough time to do it (n = 151)	20 (13%)	131 (87%)
I wanted to be vaccinated, but the vaccine was not available to me (n = 148)	2 (1.3%)	146 (99%)
I forgot about it (n = 151)	15 (10%)	136 (90%)

**Table 6:** Reasons for getting influenza vaccine, Georgia, 2022–2023.

Reasons	Yes, n (%)	No, n (%)
The flu vaccine was readily available as it was offered at my workplace (n = 162)	155 (96%)	7 (4%)
It was free (n = 156)	147 (94%)	9 (6%)
To protect my health (n = 214)	211 (99%)	3 (1%)
To protect my patient’s health (n = 176)	149 (85%)	27 (15%)
To protect my family and friends (n = 189)	176 (93%)	13 (7%)
To avoid missing work due to illness (n = 160)	119 (74%)	41 (26%)
It was recommended by the Ministry of Health and/or public health institution (n = 172)	149 (87%)	23 (13%)
Most of my colleagues were vaccinated (n = 157)	106 (68%)	51 (32%)

## Discussion

In our study of factors associated with influenza vaccine uptake among Georgian HCWs over a three-year period, we found that older HCWs were more likely to be vaccinated, while doubts about influenza vaccine safety and effectiveness were associated with lower vaccination uptake. These findings highlight not only the patterns of vaccination behaviour but also the underlying beliefs that may shape HCW decision-making. These findings, which combine three seasons of influenza vaccination history among HCWs in six Georgian hospitals, can be used to tailor messaging to emphasize influenza vaccine safety and effectiveness. In addition, the findings highlight the need to increase vaccine promotion among younger HCWs in Georgia.

Our finding that doubts about influenza vaccine safety were associated with lower vaccine uptake among HCWs has been reported in other studies conducted mostly in high-income countries. For example, in a global meta-analysis Kong et al. (2022) found that safety concerns were a common barrier to influenza vaccination among HCWs across multiple studies [23]. Likewise, a study among Lebanese physicians by Farah et al. (2025) identified vaccine safety perceptions as a key determinant influencing influenza vaccination uptake [24]. Consistent with this, a multi-country study reported that vaccine acceptance varied substantially depending on the vaccine’s perceived efficacy and safety profile [25]. These findings suggest that vaccine hesitancy among HCWs is not solely a matter of access or awareness, but reflects cognitive evaluations of risk and trust in health authorities and vaccines. Our findings, like previous studies, underscore the importance of emphasizing messages about influenza vaccine safety among HCWs. Influenza vaccine has been shown to be safe in several studies [26,27]. Strengthening post-vaccination surveillance and pharmacovigilance systems can further help address concerns about adverse events following immunization and build vaccine confidence [28]. HCWs are known to be a trusted source of information for patients [29]. Therefore, improving their awareness about vaccine safety for themselves could also help improve vaccine safety awareness among patients [30].

Similarly, doubts about influenza vaccine effectiveness were associated with lower uptake, echoing findings from post-pandemic studies, where perceived vaccine effectiveness was shown to be a strong driver of vaccine uptake decisions. For example, a German study found doubts about vaccine effectiveness as one of the most common reasons for non‑vaccination among HCWs [31]. Likewise, a study from Italy among HCWs found that confidence in the vaccine’s ability to prevent illness was the main reason for missed annual revaccination [32]. These findings indicate that HCWs’ behaviour regarding influenza vaccination is influenced by perceived utility: if the protective benefit is uncertain, motivation to vaccinate declines. From a behavioural perspective, interventions should focus on communicating the effectiveness of vaccination. Educational strategies that clearly communicate effectiveness in reducing disease severity and reducing transmission may be critical in improving coverage rates in HCWs.

Although our findings indicate that concerns about influenza vaccine safety and effectiveness were associated with lower vaccine uptake among HCWs, these perceptions represent broad constructs that may encompass different types of beliefs. Safety concerns may include evidence-based scientific reservations about vaccine adverse effects, general vaccine hesitancy, anxiety related to prior vaccination experiences, or misinformation circulating through social or professional networks. Previous qualitative research among HCWs has shown that vaccine hesitancy can stem from concerns about side effects, perceived insufficient testing of vaccines, or mistrust towards pharmaceutical companies or health authorities [29]. Future research using qualitative or mixed-methods approaches—such as in-depth interviews or focus groups—would help better characterize the specific beliefs and informational gaps underlying these perceptions among HCWs in Georgia. Such evidence could inform the development of more targeted communication strategies and vaccination promotion programs.

Age was a significant predictor of vaccine uptake in our study: HCWs in all age groups ≥ 30 years old had trends of increased odds of reported vaccination uptake compared to those in the 18–29-year group. This trend may reflect greater professional experience, heightened awareness of influenza risks, or prior exposure to institutional vaccination campaigns among older HCWs. Our finding is in line with other studies from Italy and Jordan that found that older age was significantly associated with higher influenza vaccine uptake among HCWs [33,34]. While young HCWs may be at less risk of getting severe disease from influenza virus infection, they still get ill from influenza, miss work, and they are at risk of transmitting the virus to vulnerable patients, all of which are messages that can be used to encourage influenza vaccination in this population.

The markedly lower uptake observed in Hospital B may reflect institutional factors such as differences in vaccine promotion practices, leadership engagement, workplace culture, vaccine accessibility, or local perceptions regarding vaccination. Although we did not evaluate these factors in participating hospitals, previous studies have demonstrated that organisational climate, leadership support, and institutional vaccination policies can strongly influence HCW vaccination behaviour [35,36]. Further facility-level investigations are warranted.

Our finding that there was no significant difference in influenza vaccine uptake by sex among HCWs is consistent with existing literature. For instance, Sallam et al. (2022) found that sex was not a significant predictor of influenza vaccination uptake among Jordanian HCWs [9]. Similarly, Anwar et al. (2023) reported no association between sex and vaccine uptake among HCWs in tertiary care hospitals in Bangladesh [37].

While our study population was predominantly female (90%), this gender distribution is consistent with the high proportion of women employed in nursing and allied health professions in Georgia. Annual national statistics reports in the country have consistently shown that women comprise the vast majority of the healthcare workforce; in 2022, 84.4% of physicians and 94% of nurses were women [38]. Gender-specific concerns, including perceptions related to pregnancy, fertility, or vaccine safety, could have influenced vaccination attitudes and behaviours in our survey, but could not be explored in this analysis.

While our study did not identify an association between occupational category and influenza vaccine uptake, research conducted in Germany and the United States reported significant differences in vaccination rates across different health service disciplines. The U.S. study found that physicians had significantly higher vaccination rates compared to other healthcare professionals [39]. Likewise, the German study reported that physicians had influenza vaccination rates two to three times higher than those of nurses [40].

Finally, our analysis of HCWs’ self-reported reasons for vaccination decisions underscores the role of perceived disease severity and personal health protection in decisions related to vaccination. Perception of influenza as a non-severe disease was the main reason HCWs reported for not getting the influenza vaccine in 2022–2023 season, while the desire to protect one’s own health was the main reason for getting the vaccine. These findings indicate that risk perception strongly shapes behaviour and that messages to promote vaccination should explicitly connect influenza vaccination to both individual health and patient safety outcomes. Our findings are similar to studies in Greece, the UK and Saudi Arabia, where unvaccinated HCWs reported low perception of influenza severity as a main reason for not getting vaccinated, and vaccinated HCWs reported that one of the primary motivators to get vaccinated was a desire to protect their own health [41–43]. While it is true that most young, healthy HCWs do not get severe illness from influenza virus infection, HCWs still commonly get influenza-like symptoms — including moderate illness — which poses a clear risk for transmitting infections like influenza to vulnerable patients [44].

Our study has a number of strengths. For our analysis we used data collected from a prospective cohort study among HCWs in six Georgian hospitals over three seasons, and therefore we were able to include influenza vaccination uptake data over multiple seasons. In addition, because we collected our data in a rigorously conducted prospective vaccine effectiveness study, the data were regularly checked for inconsistencies and completeness to ensure high quality.

Several limitations should be considered when interpreting our findings. First, the external validity of our study may be limited. Our analysis was conducted among HCWs in six large, secondary care hospitals in major urban centres. Therefore, these results may not be fully representative of the attitudes and vaccination behaviours of HCWs in primary care and rural medical facilities across Georgia. Our study had a high attrition rate, only 25% (403/1606) of the initial cohort providing complete data across all three years. While this longitudinal approach allowed for a robust assessment of long-term vaccination patterns, the loss of participants may have introduced selection bias; while 42% of eligible HCWs were enrolled in the prospective VE study in the six hospitals, only one-third of those HCWs were included in this analysis, as we required them to participate in all three years. Participants who remained in the study may have differed from those lost to follow-up in ways that could influence vaccination behaviour, such as greater engagement in research activities, stronger health awareness, or more positive attitudes towards vaccination. As a result, the findings may not fully represent all HCWs initially enrolled in the cohort. The causal strength of the factors we found to be associated with vaccine uptake is limited due to the observational nature of the study. While we found statistically significant associations between age, perceived vaccine safety/effectiveness, and vaccine uptake, these results demonstrate association rather than direct causality. Given the three-year study period, recall bias may have occurred, as participants may not accurately remember their vaccination history. Furthermore, social desirability bias may have influenced self-reported vaccination behaviour, potentially leading to overestimation of uptake.

Additional limitations include the relatively small sample size, as a result of which we may not have had the power to detect some true associations. Because of the limited sample size of participants with complete data across three years, we analysed receipt of influenza vaccination in at least one season as our primary outcome. As a result, individuals vaccinated once were analytically grouped with those vaccinated annually, which may limit interpretation regarding consistency of vaccination behaviour, as it does not distinguish between sporadic and consistent annual vaccination.

While our study found that HCWs at one hospital (Hospital B) had lower odds of influenza vaccination, we were not able to explore reasons that could explain the low uptake in this hospital; this is something that should be investigated in the future.

We asked participants questions about their attitudes about influenza vaccination in the Year 3 questionnaire (2023), while we used vaccination status data over the 3 previous seasons. However, the findings in our sensitivity analyses restricted to Year 3 vaccination uptake were consistent with our main findings, suggesting that temporal misclassification did not materially alter conclusions.

Finally, we did not include a comprehensive assessment of broader dimensions of vaccine hesitancy, such as trust in vaccine development and manufacturing processes, confidence in health authorities, or exposure to anti-vaccination narratives. Vaccine hesitancy is increasingly recognised as a complex and multidimensional phenomenon influenced by psychological, social, and institutional factors, particularly highlighted during the COVID-19 pandemic. While our study assessed several attitudes towards influenza vaccination, these broader determinants were beyond the scope of the survey. Future studies incorporating validated measures of vaccine hesitancy and vaccine confidence could provide additional insights into factors influencing influenza vaccine uptake among HCWs.

In conclusion, we observed that in Georgia, younger HCWs, HCWs who doubted influenza vaccine safety and influenza vaccine effectiveness, and HCWs who worked at one of the six hospitals were less likely to get vaccinated against influenza. Addressing these barriers is essential for improving vaccine uptake. Hospital-level assessments may help identify structural or organisational barriers to vaccination. Evidence from previous studies suggests that targeted educational interventions and multifaceted vaccination campaigns—such as tailored communication, on-site vaccination opportunities, and promotional activities—can increase influenza vaccination coverage among HCWs [45–47]. Implementing targeted educational programs addressing vaccine safety and effectiveness concerns, particularly among younger HCWs, could help increase vaccine uptake in Georgia. In addition, healthcare institutions in Georgia could consider expanding workplace vaccination strategies, such as on-site vaccination services, peer-led promotion, and tailored communication campaigns, to improve influenza vaccination uptake among HCWs.

## Supporting information

S1 Table Multivariable logistic regression analysis of factors associated with influenza vaccination uptake during the 2023–2024 season (Year 3 only), Georgia. (XLSX)
